# Isolation, characterization and transcriptome analysis of a novel Antarctic *Aspergillus sydowii* strain MS-19 as a potential lignocellulosic enzyme source

**DOI:** 10.1186/s12866-017-1028-0

**Published:** 2017-05-30

**Authors:** Bailin Cong, Nengfei Wang, Shenghao Liu, Feng Liu, Xiaofei Yin, Jihong Shen

**Affiliations:** The First Institute of Oceanography, State Oceanic Administration, Qingdao, 266061 People’s Republic of China

**Keywords:** Polar organisms, *Aspergillus sydowii*, Transcriptome, Lignocellulose degradation, Low temperature enzyme, Lignin peroxidase, Manganese peroxidase

## Abstract

**Background:**

With the growing demand for fossil fuels and the severe energy crisis, lignocellulose is widely regarded as a promising cost-effective renewable resource for ethanol production, and the use of lignocellulose residues as raw material is remarkable. Polar organisms have important value in scientific research and development for their novelty, uniqueness and diversity.

**Results:**

In this study, a fungus *Aspergillus sydowii* MS-19, with the potential for lignocellulose degradation was screened out and isolated from an Antarctic region. The growth profile of *Aspergillus sydowii* MS-19 was measured, revealing that *Aspergillus sydowii* MS-19 could utilize lignin as a sole carbon source. Its ability to synthesize low-temperature lignin peroxidase (Lip) and manganese peroxidase (Mnp) enzymes was verified, and the properties of these enzymes were also investigated. High-throughput sequencing was employed to identify and characterize the transcriptome of *Aspergillus sydowii* MS-19. Carbohydrate-Active Enzymes (CAZyme)-annotated genes in *Aspergillus sydowii* MS-19 were compared with those in the brown-rot fungus representative species, *Postia placenta* and *Penicillium decumbens*. There were 701CAZymes annotated in *Aspergillus sydowii* MS-19, including 17 cellulases and 19 feruloyl esterases related to lignocellulose-degradation. Remarkably, one sequence annotated as laccase was obtained, which can degrade lignin. Three peroxidase sequences sharing a similar structure with typical lignin peroxidase and manganese peroxidase were also found and annotated as haem-binding peroxidase, glutathione peroxidase and catalase-peroxidase.

**Conclusions:**

In this study, the fungus *Aspergillus sydowii* MS-19 was isolated and shown to synthesize low-temperature lignin-degrading enzymes: lignin peroxidase (Lip) and manganese peroxidase (Mnp). These findings provide useful information to improve our understanding of low-temperature lignocellulosic enzyme production by polar microorganisms and to facilitate research and applications of the novel Antarctic *Aspergillus sydowii* strain MS-19 as a potential lignocellulosic enzyme source.

**Electronic supplementary material:**

The online version of this article (doi:10.1186/s12866-017-1028-0) contains supplementary material, which is available to authorized users.

## Background

With the growing demand for fossil fuel and the severe energy crisis, lignocellulose is widely regarded as a promising, cost-effective renewable resource for bioethanol production, and the use of lignocellulose residues as a raw material has become remarkable [1–3]. However, there are numerous technological obstacles to the degradation of lignocellulose. Lignin is important organic matter that is widely present in the plant cell wall. Together with cellulose and hemicellulose, lignin forms the main component of the plant skeleton, representing the second most abundant organic regenerative resource after cellulose on earth. Since lignin and cellulose are cross-linked and lignin has complex physical and chemical properties, it represents the restrictive factor for the utilization of lignocelluloses. To effectively utilize cellulose and hemicellulose from lignocellulose raw materials, it is essential to release them from lignin bonds. Cellulose is composed of D-glucose with beta-1 and 4 glycosides, while lignin is a natural amorphous high-molecular-weight polymer. Because of the complex structure of lignin, there are no conclusive assessments of its structure to date, but a consensus has developed that the basic structure of lignin consists of phenyl propane units [4].

In their natural environments, only a small number of microorganisms are capable of degrading lignin. Lignin can be successfully implemented not only by pure cultures of particular microorganisms but also by the application of a variety of lignocellulolytic species and some non-lignocellulolytic microbes that work synergistically to break down the tough lignocellulosic structure [5–7]. The complete degradation of lignin results from the cooperation of fungi, bacteria and actinomycetes, among which fungi play the most important role. Fungi enter wood materials through hyphae while secreting extracellular enzymes that attack cellulose in the plant cell wall, resulting in the depolymerization and dissolution of lignin and cellulose. According to the type of decay caused in different lignocellulose components, the fungi can be divided into white-rot fungi, brown-rot fungi and soft-rot fungi [8]. The essence of lignin degradation consists of an oxidative process, with almost equal importance of phenol oxidase conduction. It is generally believed that lignin degradation mainly depends on four enzymes that are secreted by white-rot fungi [9]: Lac (laccase, EC 1.10.3.2) [10], LiP (lignin-peroxidase, EC 1.11.1.14) [11], Mnp (manganese peroxidase, EC 1.11.1.16) [12] and VP (versatile peroxidase, EC 1.11.1.16) [13]. Some lignin-degrading fungi do not secrete laccase, including *Phanerochete chrysosporium,* which indicates that laccase is not necessary for the degradation of lignin but could be involved in the process coordinating with other peroxidases [14]. Recently, several *Aspergillus* fungi have been shown to produce such enzymes, and additional enzymes are indispensable for complete degradation [15, 16]. Cellulase and hemicellulose are also required during lignin degradation, most of which can be categorized into the glycoside hydrolase (GH) families and carbohydrate esterase (GE) families in the Carbohydrate-Active Enzymes database (CAZy). Apart from the four above-described peroxidases, other particular enzymes also participate in or have a certain impact on lignin degradation, including cellobiose dehydrogenase (CDH,EC 1.1.99.18), glyoxal oxidase (GLOX, EC1.2.3.5), aryl alcohol oxidase (AAO,EC 1.1.3.7), glucose 1-oxidase (EC 1.1.3.4),phenol oxidase, and catalase, among others. Elena Fernández-Fueyo et al. conducted a genome analysis of *Ceriporiopsis subvermispora* and screened out all peroxidases related to lignin degradation. Their results suggests that the Lip and VP genes are not present in this strain, but two other enzymes with similar functions were identified [17]. This unexpected finding may imply that lignin degradation mechanisms vary among species.

The production of bioethanol requires the degradation of cellulose and lignin to glucose, followed by the fermentation of glucose by yeast. The temperature required for yeast fermentation is 30 °C. Low-temperature enzymes are defined as those with an optimum temperature of approximately 35 °C while maintaining a certain catalytic efficiency at 0 °C compared to the most optimum reaction temperature for cellulose ranging from 45 °C to 65 °C. Hence, if low-temperature cellulase and lignin degradation enzymes could be obtained, synchronized fermentation of the two procedures could be achieved, which would greatly simplify the production process of bioethanol and reduce costs. Although large amounts of research have been published on cellulase and lignin-degrading enzymes, few studies have investigated low-temperature lignocellulose degradation enzymes. Cecil w. Forsberg et al. reported low-temperature glucanase from the rumen thermophilic anaerobic bacteria, *Fibrobacter succinogenes* S85 [18], and another low-temperature cellulose, CelG, from the Antarctic marine thermophilic bacteria, *Pseudoalteromonas haloplanktis,* was also discovered [19]. To date, no reports on low-temperature lignin degradation enzymes have been identified.

High-throughput sequencing of transcriptomes (RNA-Seq) has provided new routes to study the genetic and functional information stored within any organism at an unprecedented scale and speed. These advances greatly facilitate functional transcriptome research in species with limited genetic resources, including many “non-model” organisms with substantial ecological or evolutionary importance [20]. Most genomics studies of lignin-degrading fungi have focused on white-rot fungi, brown-rot fungi belonging to the Basidiomycota and filamentous fungi (trichoderma, neurospora, penicillium, among others) belonging to the Ascomycota. The most widely studied white-rot fungi is *Phanerochete chrysosporium*, the genome sequence of which was published in 2004 [21]. Analyses of its genome sequence and subsequently of its transcriptional and secretary proteins have provided ample information [22–24]. Analyses of the transcriptome suggested that 545 genes or proteins were significantly altered during the lignin degradation process. Some proteins contain signal peptide and carbohydrate (CBM) domains, which may be related to the degradation of lignocelluloses. Martinez D et al. analysed the genome, transcriptome and secretome of *Postia placenta*, the most well-studied brown-rot fungus. They discovered three groups of peroxidase (LiP, Mnp and VP) and laccasein the fungal genome [25], in accordance with the inability of brown-rot fungi to degrade lignin.

This study aimed to isolate, identify and perform a transcriptome analysis of novel strains of fungi with the potential to degrade the lignocellulosic biomass isolated from the Antarctic Pole, to screen for filamentous fungi capable of producing low-temperature lignin enzymes, and to obtain lignin degradation-related enzymes through RNA-seq, with the goal of gaining insight into the mechanism underlying lignin degradation in fungi and providing a potential lignocellulosic enzyme source for industrial production.

## Methods

### Sample collection and isolation of fungi

Soil, macro-algal rot and sediment samples were collected from Ardley Island - near Fildes Peninsula, Antarctica, during the Chinese 27th Antarctic Scientific Expedition. All samples were placed in sterilized plastic bags or flasks and transported to the laboratory at 4 °C for microorganism isolation. One gram of each sample was placed in a 50-mL sterile centrifuge tube containing 10 mL of sterile distilled water and shaken at 120 rpm overnight. The tube was maintained stably overnight. Next, 10^−1^ and 10^−2^ serial dilutions of each sample suspension were spread as 0.1-mL aliquots on plates containing potato dextrose agar (PDA). The PDA plates were incubated at 12 °C for 1–2 weeks, and distinct colonies were picked and sub-cultured for further analysis.

### Phylogenetic analysis

The total DNA from 15 native fungal isolates was extracted according to the method of Gonzalez-Mendoza et al. with minor modifications [26]. Each DNA sample was amplified by PCR with Taq DNA polymerase following the manufacturer’s instructions (Tiangen, Beijing, China). Next, 2 μL DNA was used as PCR template. The primers used for amplification were ITS 1 forward (TCCGTAGGTGAACCTGCGG) and ITS 4 reverse (TCCTCCGCTTATTGATATGC). PCR amplification was performed using the following protocol: 94 °C for 5 min (1 cycle), 94 °C for 30 s, 55 °C for 30 s and 72 °C for 40 s (30 cycles), and 72 °C for 10 min(1 cycle). To confirm the quality of the PCR, the amplification products were run on 0.8% Tris acetate EDTA agarose gels, and bands were visualized by staining with ethidium bromide. The PCR products were purified using a universal DNA purification kit (Tiangen, Beijing, China), and the amplified ITS regions were sequenced (Jimei, Shanghai, China) and submitted to GenBank.

The similarities of the 15 native isolate sequences with other known species were investigated by comparisons with sequence data in the National Center for Biotechnology Information (NCBI) database using the BLASTN programme. The phylogenetic analysis was based on BioEdit multiple alignment with sequences from their closest relatives and from common fungi in the Antarctic. A phylogenetic tree based on the ITS region was constructed using MEGA5.1 software with the neighbour-joining method, and the statistical analysis utilized bootstrapping with 1000 replications.

### Enzyme assays

Fresh spores of the 15 native fungi were filtered through three layers of sterile gauze and inoculated into 150-mL flasks containing 50 mL of optimized medium for lignocellulosic enzyme production (glucose 10 g/L, ammonium sulphate 0.2 g/L, KH_2_PO_4_ 2 g/L, MgSO_4_·7H_2_O 0.5 g/L,CaCl_2_ 0.01 g/L,Vitamin B_1_ 1.0 mg/L). The flasks were incubated at 12 °C and shaken at 120 rpm. Supernatant was collected at 2d, 3d, 4d, 5d, 7d and 10d after inoculation, centrifuged at 5000 rpm to obtain cell-free samples and specific enzymatic activities were measured as follows.

Lignin peroxidase (Lip) activity was measured by the oxidation of Azure B in a reaction mixture consisting of 32 μM Azure B, 100 μM H_2_O_2_ and 50 mM sodium citrate buffer at pH 4.5 in a final volume of 1 mL. Azure B oxidation was monitored at an absorbance of 651 nm. The unit of enzymatic activity (U) was defined as an OD value reduction of 0.1 OD per mL of supernatant in 1 min, and its linear reaction time can be extended to 20 min [27].

Laccase (Lac) activity was measured using a colorimetric assay based on the oxidation of 2,2′-azino-bis(3-ethylbenzothiazoline-6-sulphonic acid (ABTS). The reaction mixture consisted of 2 mL of ABTS (0.5 mM), 1 mL of sample and 0.1 mMHAc-NaAc buffer at pH 5 and 25 °C in a final volume of 3 mL. The oxidation of ABTS was monitored from 0 to 200 s at an absorbance of 420 nm. The unit of enzymatic activity (U) was defined as 1 μM of ABTS oxidized per mL of supernatant in 1 min (ε = 3.6 * 10^4^ M^−1^ cm^−1^) [28].

Manganese peroxidase (Mnp) activity was measured by the oxidation of guaiacol in a reaction mixture added in turn to 2.9 mL of phosphate buffer (50 mM), 1 mL of H_2_O_2_ (2%), 1 mL of guaiacol (50 mM) and 0.1 mL of fermentation broth. The enzyme solution was boiled for 5 min and used as a control. The reaction system was incubated in a 34 °C water bath for 3 min immediately after addition of the fermentation broth, quickly diluted one-fold, and then monitored for phenol red oxidation at an absorbance of 465 nm once per minute a total of five times. The unit of enzymatic activity (U) was defined as △OD465/*t*_*min*_ *× 1000*.

To evaluate the effects of different temperatures and pH values on the enzymatic activity, Lip and Mnp activities were measured from 0 to 60 °C and in buffers with different pH values: Na_2_HPO_4_-KH_2_PO_4_ buffer (pH 6.0–7.5), Tris-HCl buffer (pH 7.5–9.0) and Na_2_CO_3_-NaHCO_3_ (pH 9.0–11.0). All enzymatic activities were assessed in triplicate using a GE healthcare NanoVue spectrophotometer.

### Growth measurements of *Aspergillus sydowii* MS-19

The growth of *Aspergillus sydowii* MS-19 in fermentation medium (glucose 10 g/L, ammonium sulphate 0.2 g/L, KH_2_PO_4_ 2 g/L, MgSO_4_·7H_2_O 0.5 g/L,CaCl_2_ 0.01 g/L,Vitamin B_1_ 1.0 mg/L) [29] was followed based on measurement of the dry weight of mycelia: to evaluate the effect of temperature on *Aspergillus sydowii* MS-19 growth, fermentation broth was filtered using a moderate speed qualitative filter, and mycelia were washed three times with sterile distilled water and dried at 80 °C for 3 h. Fresh spores were inoculated at a 2% inoculum concentration in six 150-mL flasks containing 50 mL of fermentation medium. The flasks were incubated at 0, 3, 10, 20, 37, and 45 °C shaken at 120 rpm for 10 days. The dry weights of the mycelia were measured once daily from day two after inoculation. To evaluate the effect of pH on *Aspergillus sydowii* MS-19 growth, fresh spores were inoculated at a 2% inoculum concentration in six 150-mL flasks containing 50 mL of fermentation medium. The flasks were incubated at pH 4, 5, 6, 7, 8 and 9 and shaken at 120 rpm at an optimized temperature (20 °C). The dry weights of the mycelia were measured upon initiation of the logarithmic growth period beginning at 7 d.

To assess the effects of lignin as the sole carbon source on growth, fermentation medium was replaced by medium containing lignin as the sole carbon source (lignin 0.3 g/L, K_2_HPO_4_ 1.0 g/L, NaCl 0.5 g/L, MgSO_4_·7H_2_O 0.3 g/L, NaNO_3_ 2.5 g/L, CaCl_2_ 0.1 g/L, FeCl_3_ 0.01 g/L, pH 7.0).

### RNA extraction and transcriptome sequencing

The *Aspergillus sydowii* MS-19 strain was grown in fermentation medium with shaking for 1 week. Mycelia were harvested by centrifugation and ground in liquid nitrogen. Total RNA samples were isolated using a standard TRIzol method, eluted in RNase-free water and stored at −80 °C until further use. Two replicates were performed for library preparation. The integrity of the total RNA was checked on an agarose gel, and its quantity and purity were determined using NanoVue (GE). mRNA was enriched from total RNA using oligo T (dT) beads, and broken into short fragments by the addition of fragmentation buffer.

cDNA was then synthesized using these fragments as template, purified, end-repaired and dA-tailed, and then ligated to sequencing adaptors. The samples were gel size-selected for the 150-bp fragment size. Size-selected adaptor-ligated cDNA was purified with an AgencourtAMPure kit (Beckman Coulter, CA, USA) and used as template for PCR amplification to create the cDNA library. The purified library were profiled using the Agilent Bio analyser and sequenced using the PE100 strategy on the IlluminaHiSeq 2000 platform to yield paired-end reads.

### Assembly and annotation of the transcriptome

Raw reads from the library were filtered to remove low-quality reads as well as adapters and poly-A/T-containing reads. The resulting clean reads were assembled to produce unigenes using the short reads assembling programme Velvet (v1.2.08) [30] and Osase (v0.2.08) [31] with default parameters. Potentially contaminated sequences were removed using BLAST.

For functional annotations, all unigene sequences were searched against Nr, eggNOG, and KEGG using BLASTX to extract predicted coding region sequences with high sequence similarity to the given unigenes along with their protein function annotations. We use the blastx with cutoff of similarity > = 80%, coverage > = 80% and evalue <1e-5. In addition, the gene ontology (GO) term for each unigeneand GO enrichment analysis was obtained using the Blast2GO programme. Pathway annotation of unigenes was performed according to the KEGG database mapping method. Unigenes were aligned to the KOG database to predict and classify possible functions.

CAZyme annotation of the *Aspergillus sydowii* MS-19 transcriptome was conducted by searching against the dbCAN database [32], which is a network resource for automatic annotation of carbohydrate active enzymes based on CAZyme tag domains for any submitted protein dataset. For each CAZyme family, a tag domain was defined by combining the search results for the conserved domain database (CDD) and published literature, and a corresponding Hidden Markov Model (HMM) was constructed. Bioedit software (v7.2.5) was used to assign genes with particular activities, including the enzyme commission (EC) number annotations.

## Results

### Fungal isolation

Six compost types were subjected to screening for the isolation and characterization of fungi. A total of 168 isolates were obtained, and their species and genera were determined based on their colony morphology and the microscopic characteristics of the spore apparatus, spore stalks and spores. Fifteen representative isolates with clear differences were selected for further analysis (Table 1). The phylogenetic tree constructed based on the ITS sequences of the 15 native isolates indicated that five isolates were members of the genus *Penicillium*, two isolates were closely related to *Pseudeurotium*, *Geomyces* and *Cladosporium*, and one isolate matched *Bionectria*, *Aspergillus*, *Aureobasidium* and an unclassified *Onygenales* (Additional file 1: Figure S1). The Antarctic region exhibited a rich diversity of fungal species.

**Table 1:** Sampling information and the representative fungal isolates

Sample name	Longitude and latitude	Number of fungi isolated	Species of fungi isolated	Representative isolates and their species	GenBank accession No.
Freshwater lake sediments (FS)	58°54′W, 62°11′S	11	*Penicillium* (5), *Geomyces* (4), *Cladosporium* (2)	FS-03 *Cladosporium cladosporioides*	JX139700
Peak umber (PU)	58°59′W, 62°11′S	14	*Penicillium* (4), *Geomyces* (5), *Aureobasidium* (5)	PU-01 *Aureobasidium pullulans*	JX675048
				PU-05 *Penicillium*	JX139707
Hill soil (HS)	58°57′W, 62°13′S	23	*Penicillium* (6), *Pseudeurotium* (5), *Geomyces* (7), *Cladosporium* (2), *Aureobasidium* (3)	HS-07 *Penicillium commune*	JX139703
				HS-11 *Pseudeurotium*	JX139704
Snow sediments (SS)	57°57′W, 62°13′S	35	*Pseudeurotium* (11), *Geomyces* (6), unclassified Onygenales (4), *Bionectria* (3), *Penicillium* (9), *Cladosporium* (1), *Aureobasidium* (1)	SS-04 *Cladosporium*	JX675049
				SS-08 unclassified Onygenales	JX139708
				SS-10 *Geomyces*	JX139709
				SS-13 *Penicillium chrysogenum*	JX139710
Macroalgae sediments (MS)	58°58′W, 62°13′ S	58	*Pseudeurotium* (9), *Geomyces* (7), unclassified Onygenales (4), *Bionectria* (6), *Penicillium* (12), *Aspergillus* (5), *Cladosporium* (8), *Aureobasidium* (7)	MS-02 *Penicillium chrysogenum*	JX139706
				MS-05 *Bionectria ochroleuca*	JX675045
				MS-17 *Pseudeurotium*	JX139705
				MS-19 *Aspergillus sydowii*	JX675047
Freshwater lake water (FW)	57°54′W, 62°11′ S	27	*Pseudeurotium* (6), *Geomyces* (4), *Penicillium* (5), *Aspergillus* (3), *Cladosporium* (3), *Aureobasidium* (6)	FW-04 *Penicillium polonicum*	JX139701
				FW-13 *Geomyces*	JX139702

Neighbour-joining tree showing the relationship between the ITS sequences from 15 Antarctic native isolates and their closest relatives as well as common fungi in the Antarctic. The bootstrap values for the neighbour-joining analysis with 1000 replicates are shown on the branches. The scale bar represents 0.05 substitutions per amino acid site.

### *Aspergillus sydowii* MS-19 is able to produce low-temperature Lip and Mnp enzymes

To test the ability of the 15 representative fungi to synthesize lignin-degrading enzymes, the strains were grown in liquid medium supplemented with the corresponding substrates. The activities of Lip, Lac and Mnp were measured. Four isolates, PU-01, SS-04, MS-05 and MS-19, showed Lip and Mnp activities (Table 2). Lac activity was not detected. Since MS-19 exhibited the highest activities of both Lip and Mnp enzymes, it was chosen as our target fungal strain in this study.

**Table 2:** Lignin-degrading enzymatic activities of the fungal isolates

Genus	Isolate No.	Highest enzymatic activity detected (U/L)	
		Lip	Mnp
*Aureobasidium*	PU-01	126.5	113.8
*Cladosporium*	SS-04	136.8	126.4
*Bionectria*	MS-05	154.2	132.6
*Aspergillus*	MS-19	182.6	159.7

**Fig. 1:**
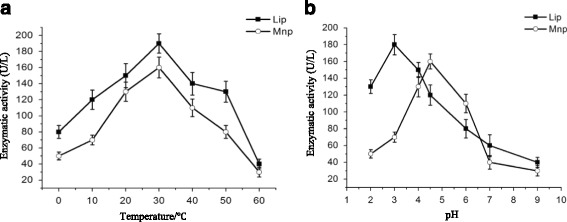
Enzymatic assay of Lip and Mnp produced by MS-19 at different temperatures and pH values. **a** Enzymatic activities of Lip and Mnp synthesized by MS-19 at different temperatures. **b** Enzymatic activities of Lip and Mnp synthesized by MS-19 at different pH values

Lip and Mnp activities were measured at different temperatures and pH values to identify the optimum condition for enzymes produced by MS-19. Lip and Mnp maintained a certain activity at 0 °C and gradually increased with temperature, showing the highest activity at 30 °C and declining sharply above this temperature (Fig. 1a). Since low-temperature enzymesare defined to have an optimal temperature of approximately 35 °C and to maintain a certain catalytic efficiency at 0 °C, the Lip and Mnp enzymes synthesized by MS-19 were considered low-temperature enzymes. The optimal pH for Lip and Mnp was 3.0 and 4.5, respectively. Lip was found to have sufficiently high activity between pH 2.0–4.0, while Mnp showed high activity between pH 4.0–6.0 (Fig. 1b).

**Fig. 2:**
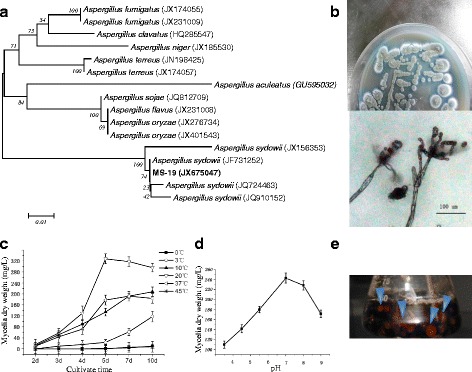
Phylogeny and growth profile of Aspergillus sydowii MS-19. **a** Neighbour-joining tree showing the relationship between ITS sequences from MS-19 and its 15 closest relatives. Bootstrap values for the neighbour-joining analysis with 1000 replicates are shown on the branches. The scale bar represents 0.01 substitutions per amino acid site. **b** The morphological and microscopic characteristics of Aspergillus sydowii MS-19. **c** Time course of Aspergillus sydowii MS-19 growth at different temperatures. **d** Growth of Aspergillus sydowii MS-19 at different initial pH values. **e** Mycelial pellets were observed when Aspergillus sydowii MS-19 was cultured in medium with lignin as the sole carbon source. Blue arrowheads indicate white mycelial pellets in growth medium

BLAST searches demonstrated that MS-19 was highly similar to *Aspergillus* (Additional file 1: Figure S1). To further identify the phylogeny of MS-19, a phylogenetic tree was constructed based on the ITS sequences of MS-19 and its 15 closest relatives. MS-19 displayed 99% identity to *Aspergillus sydowii* (Fig. 2a). Combined with the morphological and microscopic characteristics of MS-19 (Fig. 2b), the genus of the MS-19 isolate was identified as *Aspergillus. sydowii*.

*Aspergillus sydowii* MS-19 could grow in the temperature range from 3 to 37 °C. Its growth was observed at temperatures as low as 3 °C. The growth rate was maximal at 20 °C and pH 7 (Fig. 2c and d). A white mycelial pellet could be observed after 7 days of culturing at 20 °C, demonstrating that *Aspergillus sydowii* MS-19 could utilize lignin as a sole carbon source (Fig. 2e).

### Summary of the RNA-seq dataset

To obtain an overview of the *Aspergillus sydowii* MS-19 transcriptome, the poly (A)-enriched mRNA sample was subjected to high-throughput IlluminaHiSeq sequencing, resulting in 18,453,231 reads with an average length of 101 nt. Assembler Velvet and Oases was employed to complete the assembly and cluster of the *Aspergillus sydowii* MS-19 transcriptome using default parameters. From the Velvet assembly were obtained 72,387contigs with a total length of 23,862,098 nt. Oases continued to generate longer transcripts, resulting in 27,600 transcripts with a length of 21,976,433 nt. Additional sequence cluster analyses were conducted among all transcript sequences, generating 11,269 unigenes with a mean size of 1130 nt (Table 3).

**Table 3:** Output statistics for the sequencing and assembly

Sample	*Aspergillus sydowii* MS-19
Raw Reads	28,267,863
Clean Reads	18,453,231
Read size (nt/read)	101
Total nucleotides (nt)	3,690,646,200
Contigs	
Number of contigs	72,387
Mean size of contigs	330
Length of all contigs (nt)	23,862,098
Transcripts	
Number of transcripts	27,600
Mean size of transcripts	796
Length of all transcripts (nt)	21,976,434
Unigenes	
Number of unigenes	11,269
Mean size of unigenes	1130
Length of all unigenes	12,735,577

### GO and KOG classification

**Fig. 3:**
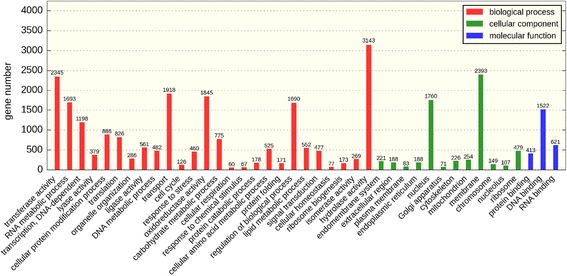
Histogram of the gene ontology classification

**Fig. 4:**
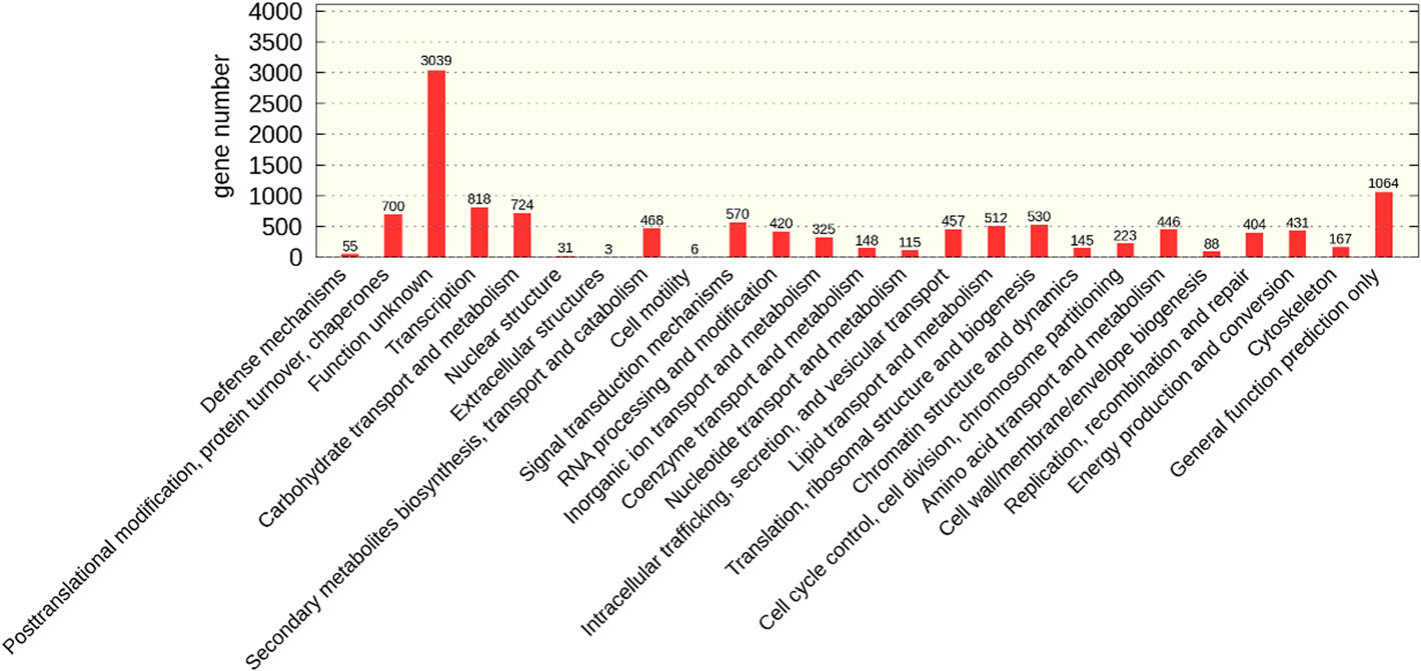
Histogram of the KOG classification. The x-axis indicates 25 KOG groups, and the y-axis indicates the number of genes annotated in the current group

**Fig. 5:**
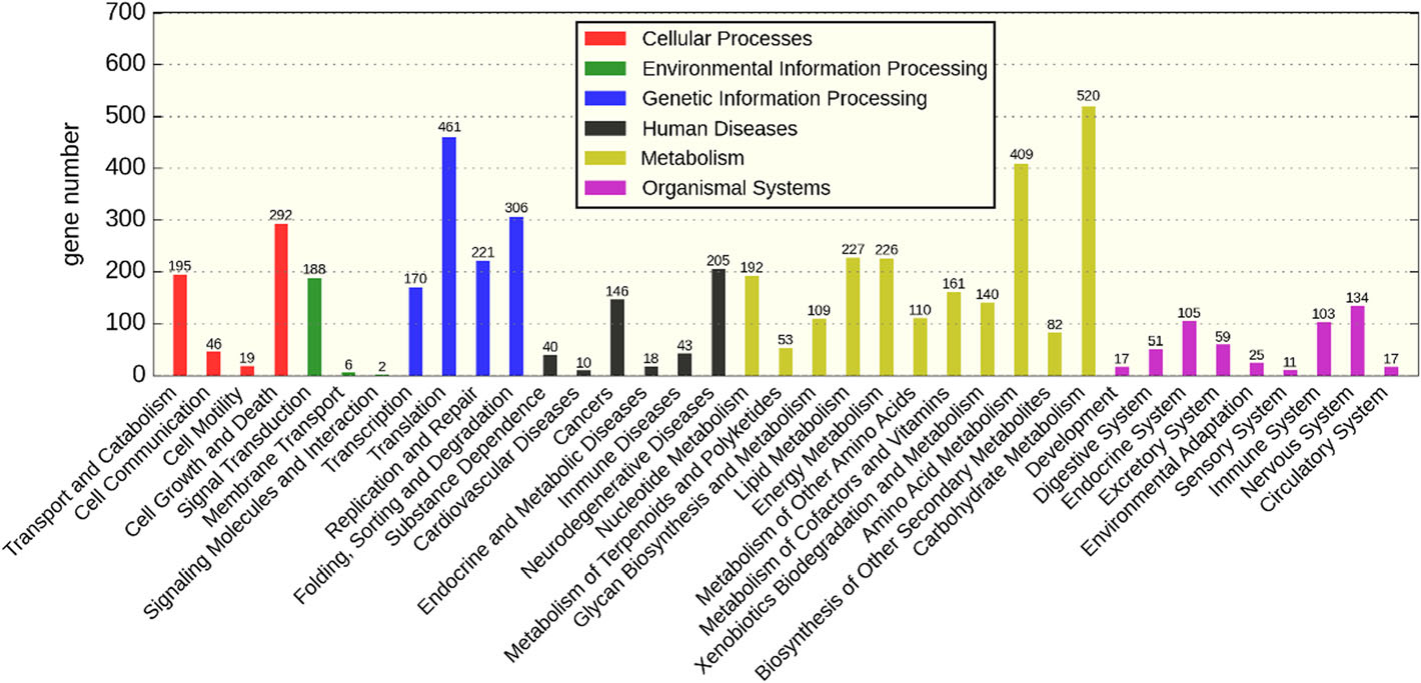
Histogram of the KEGG classification

GO assignments were used to classify the unigene functions of *Aspergillus sydowii* MS-19. Based on the sequence homology, 8199 unigenes were categorized into 55 functional groups. In terms of biological process, the majority of the unigenes were involved in “hydrolase activity” (3143 members), “transferase activity” (2345 members) and “transport” (1918 members). For the cellular component, the majority of the unigenes were involved in “membrane” (2393 members), “nucleus” (1760 members) and “ribosome” (479 members). The investigation of molecular functions revealed that most unigenes were involved in “DNA binding” (1522 members), “RNA binding” (621 members) and “structural molecule activity” (515 members) (Fig. 3). To further evaluate the completeness of the transcriptome and the effectiveness of the annotation process, the annotated sequences were screened for genes involved in KOG classifications. In total, among17767 nr hits, 11,192 sequences had KOG classifications. Among the 25 KOG categories, the cluster for “function unknown” represented the largest group (3,039members), followed by “general function prediction” (1064 members) and “transcription” (818 members). The following categories represented the smallest groups: Extracellular structures (3 members); Cell motility (6 members) and Nuclear structure (31 members) (Fig. 4). To identify the biological pathways that were active in *Aspergillus sydowii* MS-19, we mapped all the unigenes to the reference canonical pathways in KEGG, and we found that a total of 3533 sequences could be assigned to 39KEGG pathways. The most representative pathways by the unigenes were “metabolism pathways” (2229 members), “genetic information processing” (1158 members), “cellular processes” (552 members), Organismal Systems (522 members) and “environmental information processing” (196 members) (Fig. 5). These annotations provide a valuable resource for investigating specific processes, functions and pathways in *Aspergillus sydowii* MS-19.

GO has three ontologies: molecular function, cellular component and biological process, indicating the GO functional classification annotation and the number of unigenes in each category. The x-axis indicates the term description, and the y-axis indicates the number of genes annotated in the current group (GO terms with less than 50 annotated genes are not shown).

The KEGG pathway annotation provides a mapping of the transcriptomic dataset to the KEGG pathway maps for biological interpretation of higher-level systemic functions. It indicates the KEGG pathway classification annotation and the percentage of each pathway.

Since our main concern was material degradation, we focused on sequences that participate in carbohydrate metabolism and xenobiotic biodegradation pathways (Additional file 2: Table S2). Most fungi adopt the Embden-Meyerhof-Parnas pathway (EMP) for carbohydrate metabolism, while a few fungi such as red yeast utilize the pentose phosphate pathway (HMP). Annotation of the *Aspergillus sydowii* MS-19 transcriptome revealed enzymes that play important roles in the EMP, such as hexokinase, glucose-6-phosphate isomerase, fructose-phosphate kinase phosphatases, glyceraldehyde-3-phosphate dehydrogenase, pyruvate kinase, acetyl coenzyme synthetase and 6-phosphate glucose isomerase, indicating that the EMP is the main carbohydrate metabolism pathwayin *Aspergillus sydowii* MS-19.

Regarding the annotated unigenes associated with xenobiotic biodegradation, some enzymes are capable of metabolizing the benzene ring structure. These enzymes may contribute to lignin degradation, which is a class of polymer composed of phenyl propane as the structural unit. Relevant benzene ring-metabolizing enzymes are as follows: 2-haloacid dehalogenase, aldehyde dehydrogenase and S-(hydroxymethyl) glutathione dehydrogenase related to the degradation of chloroalkane and chloroalkene; catechol 1,2-dioxygenase and carboxymethylenebutenolidase related to the degradation of toluene and chlorobenzene; phenylacetate 2-hydroxylase, fumarylacetoacetase, fumarylacetoacetase, amidase, nitrilase and 3-hydroxyphenylacetate 6-hydroxylase related to the degradation of styrene; salicylate hydroxylase related to the degradation of dioxin;glutathione S-transferase; and S-(hydroxymethyl) glutathione dehydrogenase in the cytochrome P450 superfamily related to xenobiotic metabolism, among others. These annotations indicate that *Aspergillus sydowii* MS-19 not only has the ability to metabolize xenobiotics and environmental pollutants but also may degrade lignin.

### CAZyme expression profiles

**Fig. 6:**
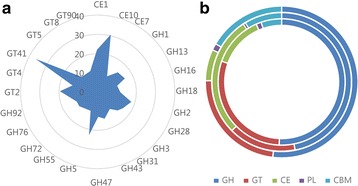
Comparison of the numbers of unigenes belonging to CAZy families (**a**) in *Aspergillus sydowii* MS-19 and (**b**) in *Aspergillus sydowii* MS-19**,** *Postia placenta* and *Penicillium decumbens.* **a** The numbers of unigenes belonging to particular CAZy families in *Aspergillus sydowii* MS-19, only CAZy families containing 10 or more unigenes are shown. **b** Distribution of various CAZymes in *Aspergillus sydowii* MS-19 (outer ring)**,** *Postia placenta* (middle ring) and *Penicillium decumbens* (outer ring). GH, glycoside hydrolase; GT, glycosyltransferase; CE, carbohydrate esterase; PL, polysaccharide lyase; CBM, carbohydrate-binding module

Using the CAZy database, a total of701 unigenes were annotated, including 355 glycoside hydrolase (GH), 208 glycosyltransferase (GT), 9 polysaccharide lyase (PL), 92 carbohydrate esterase (CE) and 37 carbohydrate combined structure domain (CBM) proteins (Fig. 6a). The most representative families were GT41 (38 members), CE10 (31 members) and GH5 (24 members).

There were 37 genes encoding proteins containing the cellulose-binding domain (CBM), which may assist in lignocellulose-degrading enzyme attachment to the surface of cellulose and thus facilitate co-degradation of the natural cellulose-hemicellulose network.

CAZyme-annotated genes in *Aspergillus sydowii* MS-19 and the brown-rot fungi representative species, *Postia placenta* and *Penicillium decumbens*, were compared (Fig. 6, b). The total number of CAZymes and the number of CAZymes in the GH and GT classes of *Aspergillus sydowii* MS-19 were much higher than in the other two.

The CAZymes in *Aspergillus sydowii* MS-19 related to lignocellulose degradation are listed in Table 4. There were 17 cellulase (eight cellobiohydrolase and nine endo-1,3-beta-glucanase) and 19 feruloyl esterase-encoding genes. In addition, 13 chitinase genes were annotated, and the chitin*-*degrading ability of *Aspergillus sydowii* MS-19 is subject to verification.

**Table 4:** CAZymes in *Aspergillus sydowii* MS-19 related to lignocellulose degradation

CAZyme	Number	Locus
cellobiohydrolase	8	Locus_1390,Locus_2944,Locus_4414,Locus_6027,Locus_7420,Locus_15666,Locus_22469,Locus_24089
endo-1,3-beta-glucanase	9	Locus 526,Locus 1086,Locus 1179, Locus 2141,Locus 3549,Locus 7936,Locus 21,439,Locus 22,695,Locus 24,357
β-glucosidase	57	Locus_10259, Locus_10547, Locus_10915, Locus_1108, Locus_11132, Locus_114, Locus_11458, Locus_12029, Locus_13235, Locus_13894, Locus_13917, Locus_14024, Locus_14178, Locus_14313, Locus_14583, Locus_14893, Locus_1560, Locus_16303, Locus_16488, Locus_16896, Locus_16926, Locus_17760, Locus_17789, Locus_17844, Locus_1900, Locus_1912, Locus_1939, Locus_19428, Locus_20804, Locus_2085, Locus_21268, Locus_21724, Locus_21959, Locus_22387, Locus_23309, Locus_23358, Locus_23698, Locus_25281, Locus_26343, Locus_27, Locus_2734, Locus_3147, Locus_3781, Locus_3913, Locus_4347, Locus_4646, Locus_4820, Locus_5135, Locus_5202, Locus_5954, Locus_6125, Locus_6476, Locus_6499, Locus_7420, Locus_8287, Locus_931, Locus_9711
α- glucosidase	24	Locus_11276, Locus_12706, Locus_13235, Locus_14736, Locus_18, Locus_18186, Locus_18361, Locus_19137, Locus_19676, Locus_2131, Locus_22493, Locus_23154, Locus_24653, Locus_3331, Locus_354, Locus_3592, Locus_4621, Locus_5377, Locus_5565, Locus_5981, Locus_8096, Locus_856, Locus_8769, Locus_9406
endo-1,4-beta-xylanase	2	Locus_21348,Locus_14631
beta-xylosidase	37	Locus_10259, Locus_11943, Locus_13192, Locus_13650, Locus_13917, Locus_14313, Locus_14583, Locus_15712, Locus_16267, Locus_16303, Locus_16896, Locus_17760, Locus_18132, Locus_1844, Locus_1912, Locus_1915, Locus_2085, Locus_21472, Locus_21724, Locus_23358, Locus_25098, Locus_25281, Locus_3147, Locus_3913, Locus_4820, Locus_5135, Locus_5202, Locus_5954, Locus_6125, Locus_6476, Locus_7051, Locus_8287, Locus_8971, Locus_9039, Locus_931, Locus_9711, Locus_9815
beta-mannosidase	42	Locus_10547, Locus_10915, Locus_1108, Locus_11132, Locus_114, Locus_11458, Locus_11470, Locus_12029, Locus_1319,Locus_13235, Locus_13828, Locus_13894, Locus_14024, Locus_14178, Locus_14381, Locus_14823, Locus_14893, Locus_1560, Locus_16488, Locus_16926, Locus_17112, Locus_17789, Locus_17844, Locus_1862, Locus_1900, Locus_19428, Locus_20143, Locus_20804, Locus_20846, Locus_21268, Locus_21959, Locus_21986, Locus_22387, Locus_23309, Locus_23698, Locus_2531, Locus_26343, Locus_3619, Locus_4347, Locus_4646, Locus_6354, Locus_6499
galactosidase	63	Locus_10547, Locus_1086, Locus_10915, Locus_1108, Locus_11372, Locus_11458, Locus_11470, Locus_1179, Locus_11943, Locus_12029, Locus_1319, Locus_13192, Locus_13235, Locus_13565, Locus_13650, Locus_13828, Locus_13915, Locus_14024, Locus_14381, Locus_14823, Locus_15680, Locus_15712, Locus_16267, Locus_16926, Locus_17112, Locus_17184, Locus_17789, Locus_17844, Locus_18132, Locus_1844, Locus_1862, Locus_1915, Locus_20143, Locus_20804, Locus_20846, Locus_21268, Locus_2141, Locus_21,439, Locus_21465, Locus_21472, Locus_21986, Locus_22270, Locus_22,695, Locus_24,357, Locus_24863, Locus_25098, Locus_2531, Locus_26343, Locus_3549, Locus_3619, Locus_3930, Locus_4347, Locus_4646, Locus_526, Locus_6354, Locus_6561,Locus_7051, Locus_7512, Locus_7936, Locus_8060, Locus_8971, Locus_9039, Locus_9815
feruloyl esterase	19	Locus_10175, Locus_11024, Locus_11435, Locus_12982, Locus_17024, Locus_17982, Locus_18274, Locus_2117, Locus_3617, Locus_365, Locus_4119, Locus_4156, Locus_5080, Locus_5575, Locus_5779, Locus_6122, Locus_7841, Locus_8559, Locus_858
pectate lyase	6	Locus_10429, Locus_13318, Locus_16762, Locus_17762, Locus_19898, Locus_4613
acetylesterase	6	Locus_15519, Locus_15609, Locus_18146, Locus_18619, Locus_20150, Locus_21291
chitinase	13	Locus_1056, Locus_12689, Locus_12831, Locus_14176, Locus_1429, Locus_16340, Locus_1636, Locus_1648, Locus_18421, Locus_21977, Locus_2206, Locus_3516, Locus_483

**Fig. 7:**
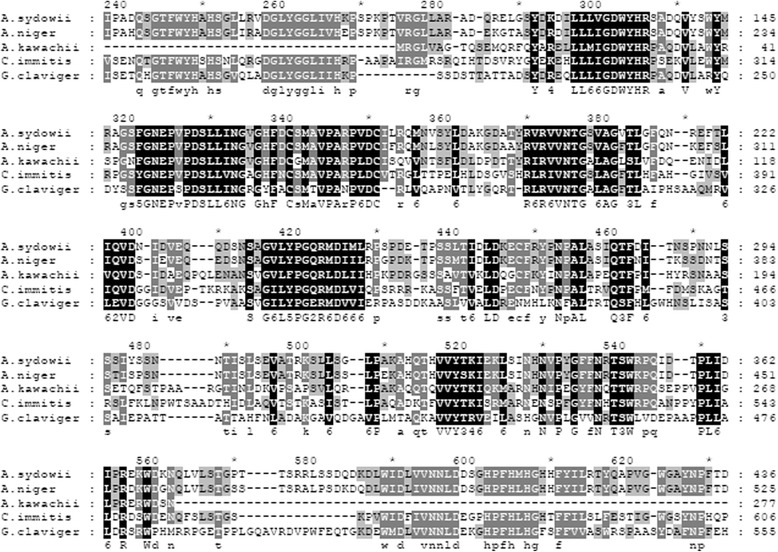
Alignment of the amino acid sequence of *Aspergillus sydowii* MS-19 (A.sydowii) laccase-like gene with the laccase sequences from four other fungal species. The species selected for the alignment were *Coccidioides immitis* (EAS35187, C.immitis), *Grosmannia clavigera* (EFX01145, G claviger), *Aspergillus kawachii* (GAA87354, A.kawachii), and *Aspergillus niger* (XP_001389525, A.niger)

Remarkably, one sequence annotated as laccase, which can degrade lignin, was found at Locus_6288. The BLAST results showed that the nucleic acid sequence homology between this sequence and the laccase sequences from *Coccidioides immitis* (EAS35187), *Grosmannia clavigera* (EFX01145), *Aspergillus kawachii* (GAA87354), and *Aspergillus niger* (XP_001389525) was 52%, 40%, 54% and 53%, respectively (Fig. 7).

**Fig. 8:**
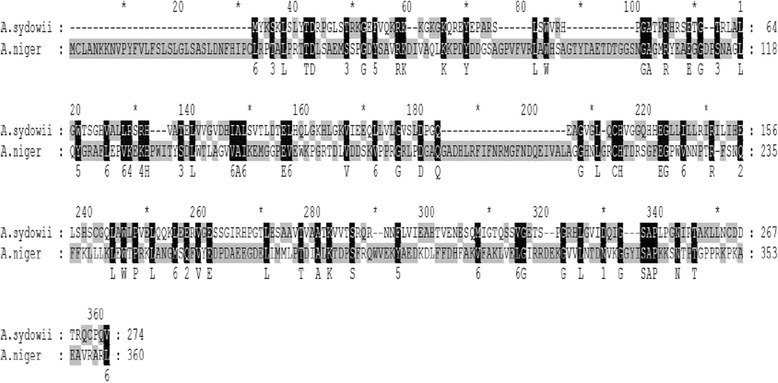
Alignment of the amino acid sequences of *Aspergillus sydowii* MS-19 haem-binding peroxidase-like gene with the same gene from *Aspergillus niger*

Three peroxidase sequences sharing a structure similar to typical lignin peroxidase and manganese peroxidase were found and annotated as haem-binding peroxidase, glutathione peroxidase and catalase-peroxidase. Among them, the haem-binding peroxidase annotated sequence (Locus_2354) displayed the highest similarity, and the BLAST results showed a nucleic acid sequence homology of 75% between this sequence and *Aspergillus niger* CBS 513.88 haem-binding peroxidase (Fig. 8). It is speculated that this gene may have different names but similar functionalities.

## Discussion

Polar microorganisms have important value in scientific research as well as in application development since they provide novelty, uniqueness and diversity. An increasing number of people are now focused on the study and application of low-temperature enzymes. In recent years, due to the shortage of energy sources, utilization of lignocellulosic material to produce liquid fuels and chemicals has become an important alternative approach for supporting the sustainable development of human society. Low-temperature lignocellulosic enzymes also show great potential applications in industrial production [33]. In this study, we isolated and identified a fungus, *Aspergillus sydowii* MS-19, from the Antarctic region that is able to produce the low-temperature enzymes, Lip and Mnp. We also adapted high-throughput sequencing technology to characterize the transcriptome features and genes encoding CAZymes in *Aspergillus sydowii* MS-19. This work will provide useful information to facilitate our understanding of low-temperature lignocellulosic enzyme production by polar microorganisms, as well as further research and the application of a novel Antarctic *Aspergillus sydowii* strain MS-19 as a potential source of lignocellulosic enzymes.

RNA-seq reads of *Aspergillus sydowii* MS-19 were assembled and subsequently clustered into 11,269unigenes, among which most of the sequences were annotated against three databases. The Blast2GO framework was used to analyse the annotation results based on transcriptomic research. Among the unigenes, 3.82% of 11,269 accounted for catabolism processes involved in lignin degradation and glycometabolism. Most of the unigenes generated products inside the cell, whereas a few generated extracellular products, potentially due to the different cultivating conditions. In many lignocellulose-degrading microorganisms, lignocellulosic enzyme secretion issignificantly enhanced following the addition of particular inducers were added [34, 35]. Among the MFs (molecular functions) of the annotated sequences, 10.48%, 14.12%, 1.14% were responsible for DNA-binding, ion binding and enzyme binding, respectively, which may be related to gene transcription regulation and protein-folding transportation, and can be used for further studies of the synthesis and regulatory mechanisms of lignocellulosic enzymes [36]. The largest portion of annotated unigenes against the KEGG database was associated with metabolic pathways. Enzymes involved in these pathways could metabolize cellulose, hemicellulose, and starch, and they were also carbohydrate-active enzymes.

Cellobiohydrolase and endo-1,4-beta-xylanase ensured effective lignocellulose degradation in *Aspergillus sydowii* MS-19 [37, 38]. Enzymes such as 2-hydrochloric acid halide can degrade the benzene ring. Since lignin is a polymer composed of a phenyl propane structural unit, its expression in *Aspergillus sydowii* MS-19 suggested a great potential ability to degrade xenobiotic and environmental pollutants, even lignin [39]. Mariana et al. have reported that *Aspergillus sydowii* Gc12 isolated from sponge can secrete peroxidase to catalyse loop-opening of benzyl glycidyl ether (a lignin monomer analogue) [40].

An interesting sequence located in Locus_6288 was presumed to perform a similar function to laccase. The sequence identity between Locus_6288 in *Aspergillus sydowii* MS-19 and laccase in *Aspergillus niger* ATCC 1015 reached 53%. However, the enzyme activity assay of *Aspergillus sydowii* MS-19 in this study did not detect laccase activity. These contradictory results may be due to the small amount and low activity of laccase secreted extracellularly. The laccase activity of *Aspergillus sydowii* MS-19 could by further analysed by increasing the enzyme concentration.

We also observed that three sequences, annotated as haem-binding peroxidase, glutathione peroxidase and catalase-peroxidase, were similar to typical lignin peroxidase and manganese peroxidases, all of which had haem binding sites based on a multi-alignment analysis. This result suggests the presence of peroxidase sequences with different names that perform similar functions in *Aspergillus sydowii* MS-19. Elena Fernández-Fueyo et al. [17] screened all peroxidase-encoding genes associated with lignin degradation in *Ceriporiopsis subvermispora*. Instead of the LiP and VP gene, they found two other peroxidases with similar functions. There is a relatively conserved tryptophan residue exposed outside the protein tertiary structure in the two peroxidases, which plays an important role in electron transportation during lignin degradation. Whether enzymes encoded by haem-binding peroxidase genes participate in *Aspergillus sydowii* MS-19 lignin degradation remain to be elucidated.

At present, *Phanerochete chrysosporium* is the most comprehensive studied. It shows the best enzyme activity in ligninase research field. According to the unit definition and the measurement method of ligninase proposed by Archibald [41] and Gleen [42], the optimized Lip and Mnp activities of *Phanerochete chrysosporium* may reach 300 ~ 1000 U/L, and the optimal enzyme activity temperature is as high as 40 ~ 45 °C. The novel Antarctic *Aspergillus sydowii* MS-19 we obtained in this study has low temperature ligninase activity, its optimal enzyme activity is 30 °C. The maximum enzyme activity of Lip and Mnp are 182.6 U/ L and 159.7 U/L for *Aspergillus sydowii* MS-19, which are lower than that of *Phanerochete chrysosporium*. This can be attributed to the most suitable conditions for enzyme production, influencing factors and mutagenesis for *Phanerochete chrysosporium* that have been studied thoroughly and higher enzyme activity is obtained. Low temperature enzyme such as *Aspergillus sydowii* MS-19 has advantages over *Phanerochete chrysosporium* in bio energy applications field in our study. The production of bio ethanol requires degradation of cellulose and lignin to glucose, followed by the fermentation of glucose by yeast. The reaction temperature of conventional cellulase and ligninase are between 45 ~ 65 °C, while the temperature required for yeast fermentation is 30 °C. Thus the two processes are separate, and with the increase of reaction time, the concentration of glucose is feedback inhibited. It not only affects the production efficiency, but also greatly increases the cost of bioethanol production. Hence, if low temperature cellulase and lignin degradation enzymes is obtained, synchronization fermentation of the two procedures would achieve. This will greatly simplify the production process of bio ethanol with lower cost. Excess glucose can also be used for yeast fermentation medium. Synchronization fermentation can also reduce the concentration of glucose, remove feedback inhibition and improve the yield of ethanol. We have obtained the low temperature cellulase (optimal enzyme activity temperature 30 °C). Meanwhile, the isolated strain in this study has the ability of producing low temperature ligninase. For further research, these results will provide a solid theoretical basis for the industrial production of bioethanol, and provide a new way of production. In a word, because of the huge difference of reaction temperature, there is no direct comparability between the strains of *Aspergillus sydowii* MS-19 and industrialized strains.

## Conclusions

In the present investigation, a fungus, *Aspergillus sydowii* MS-19, with the potential for lignocellulose degradation was screened out and isolated from the Antarctic region. We measured the growth profile of *Aspergillus sydowii* MS-19 and found that it could utilize lignin as a sole carbon source. We also verified its ability to synthesize low-temperature lignin peroxidase (Lip) and manganese peroxidase (Mnp) enzymes, the properties of which were also investigated. High-throughput sequencing was employed to identify and characterize the transcriptome of *Aspergillus sydowii* MS-19. Carbohydrate-Active Enzymes (CAZyme)-annotated genes in *Aspergillus sydowii* MS-19 were compared with those in the brown-rot fungi representative species, *Postia placenta* and *Penicillium decumbens*. One sequence annotated as laccase, which can degrade lignin, and three peroxidase sequences sharing a similar structure with typical lignin peroxidase and manganese peroxidase were obtained. The novel Antarctic *Aspergillus sydowii* strain MS-19 could be utilized as a potential source of lignocellulosic enzymes.

## Additional files

Additional file 1: Figure S1. Phylogeny of Antarctic fungal isolates. Neighbour-joining tree showing the relationship between the ITS sequences from 15 Antarctic native isolates and their closest relatives as well as common fungi in Antarctic. The bootstrap values of the neighbor-joining analysis with 1000 replications are shown on the branches. The scale bar represents 0.05 substitutions per amino acid site. The isolated strains can be classified into 4 classes: *Eurotiomycetes, Leotiomycetes, Sordariomycetes and Dothideomycetes*. All the 18S/ITS sequences of isolated fungus have been submitted to GenBank and the GenBank accession No. can be available in the brackets. (PPTX 76 kb)

Additional file 2: Table S2. Annotated unigenes associated with carbohydrate metabolism and xenobiotic biodegradation. Annotated unigenes, pathways and corresponding members number associated with carbohydrate metabolism and xenobiotic biodegradation after the CAZyme annotation of *A. sydowii* MS-19 transcriptome. (DOCX 18 kb)

## Abbreviations

CAZyme: Carbohydrate-active enzymes
CBM: Carbohydrate-binding modules
CE: Carbohydrate esterase
GH: Glycoside hydrolase
GO: Gene ontology
GT: Glycosyltransferase
ITS: Internal transcribed spacer
KEGG: Kyoto encyclopaedia of genes and genomes
Lac: Laccase
Lip: Lignin peroxidase
Mnp: Manganese peroxidase
PL: Polysaccharide lyase
VP: Versatile peroxidase
