# Supplementary material for: Isolation, characterization and transcriptome analysis of a novel Antarctic Aspergillus sydowii strain MS-19 as a potential lignocellulosic enzyme source
Source: BMC Microbiol. 2017 May 30;17:129. doi: 10.1186/s12866-017-1028-0 (PMC5450402; doi:10.1186/s12866-017-1028-0)
Supplement: Supplementary file 1 — Phylogeny of Antarctic fungal isolates. Neighbour-joining tree showing the relationship between the ITS sequences from 15 Antarctic native isolates and their closest relatives as well as common fungi in Antarctic. The bootstrap values of the neighbor-joining analysis with 1000 replications are shown on the branches. The scale bar represents 0.05 substitutions per amino acid site. The isolated strains can be classified into 4 classes: Eurotiomycetes, Leotiomycetes, Sordariomycetes and Dothideomycetes. All the 18S/ITS sequences of isolated fungus have been submitted to GenBank and the GenBank accession No. can be available in the brackets. (PPTX 76 kb) [file 12866_2017_1028_MOESM1_ESM.pptx]

## Slide 1
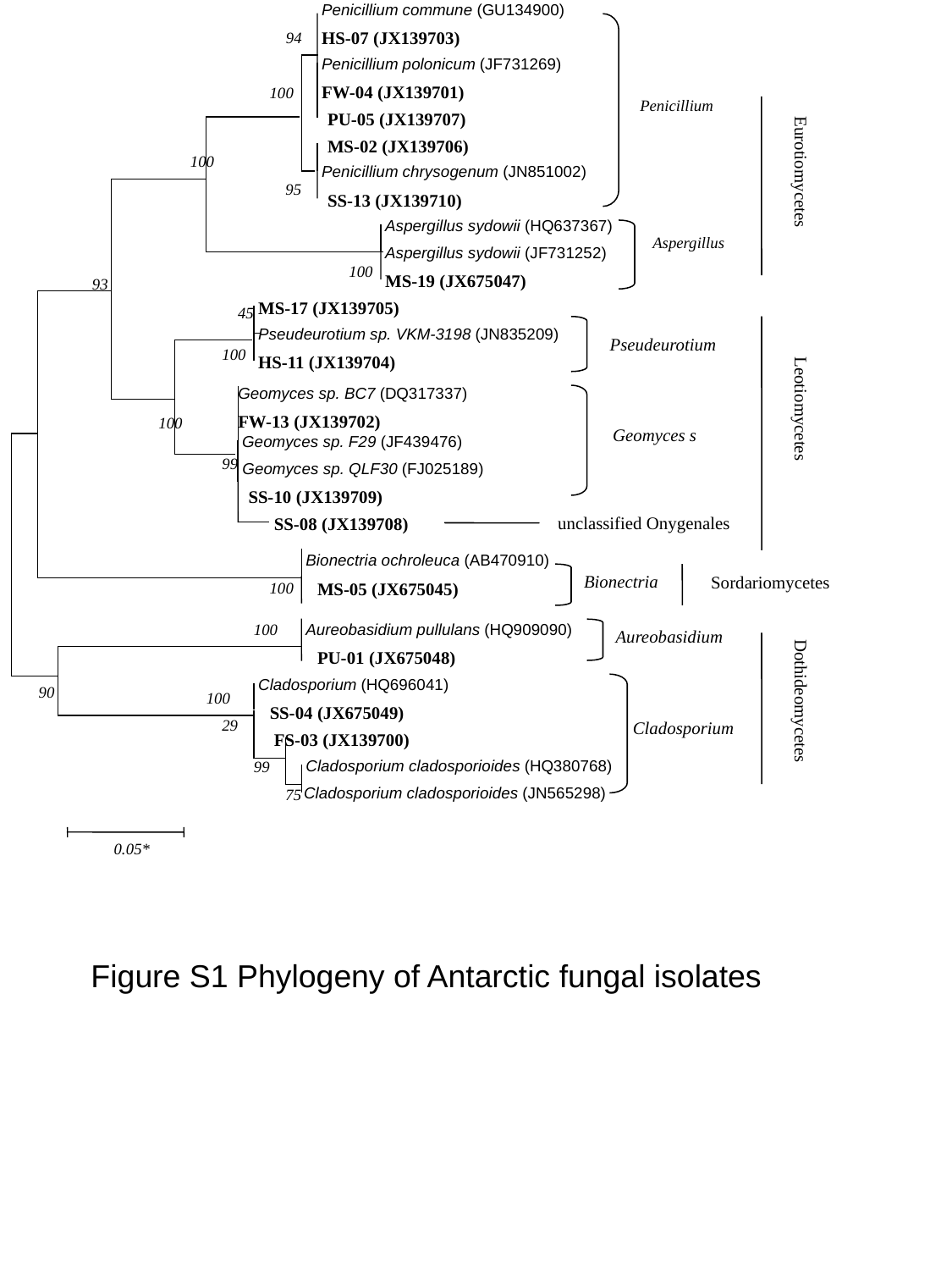

Penicillium commune (GU134900)
 HS-07 (JX139703)
94
 Penicillium polonicum (JF731269)
 FW-04 (JX139701)
100
 PU-05 (JX139707)
Eurotiomycetes
 MS-02 (JX139706)
100
 Penicillium chrysogenum (JN851002)
95
 SS-13 (JX139710)
 Aspergillus sydowii (HQ637367)
Aspergillus
 Aspergillus sydowii (JF731252)
100
 MS-19 (JX675047)
93
 MS-17 (JX139705)
45
 Pseudeurotium sp. VKM-3198 (JN835209)
100
Leotiomycetes
 HS-11 (JX139704)
Geomyces sp. BC7 (DQ317337)
FW-13 (JX139702)
100
 Geomyces sp. F29 (JF439476)
99
 Geomyces sp. QLF30 (FJ025189)
 SS-10 (JX139709)
 SS-08 (JX139708)
 Bionectria ochroleuca (AB470910)
Bionectria
100
MS-05 (JX675045)
100
 Aureobasidium pullulans (HQ909090)
Aureobasidium
Dothideomycetes
PU-01 (JX675048)
 Cladosporium (HQ696041)
90
100
SS-04 (JX675049)
Cladosporium
29
 FS-03 (JX139700)
 Cladosporium cladosporioides (HQ380768)
99
 Cladosporium cladosporioides (JN565298)
75
0.05*
Penicillium
Pseudeurotium
Geomyces s
unclassified Onygenales
Sordariomycetes
Figure S1 Phylogeny of Antarctic fungal isolates
