# Supplementary material for: Isolation, characterization and transcriptome analysis of a novel Antarctic Aspergillus sydowii strain MS-19 as a potential lignocellulosic enzyme source
Source: BMC Microbiol. 2017 May 30;17:129. doi: 10.1186/s12866-017-1028-0 (PMC5450402; doi:10.1186/s12866-017-1028-0)
Supplement: Supplementary file 2 — Annotated unigenes associated with carbohydrate metabolism and xenobiotic biodegradation. Annotated unigenes, pathways and corresponding members number associated with carbohydrate metabolism and xenobiotic biodegradation after the CAZyme annotation of A. sydowii MS-19 transcriptome. (DOCX 18 kb) [file 12866_2017_1028_MOESM2_ESM.docx]

Table S1 Annotated unigene associated with carbohydrate metabolism and xenobiotic biodegradation

|  | Pathway | Annotated unigene | members |
| --- | --- | --- | --- |
| Carbohydrate  Metabolism | Glycolysis / Gluconeogenesis | HK; hexokinase | 7 |
|  |  | GPI;glucose-6-phosphateisomerase | 2 |
|  |  | PFK;6-phosphofructokinase | 4 |
|  |  | FBP;fructose-1,6-bisphosphataseI | 6 |
|  |  | ALDO;fructose-bisphosphatealdolase, class I | 4 |
|  |  | triosephosphate isomerase (TIM) | 8 |
|  |  | GAPDH, gapA; glyceraldehyde 3-phosphate dehydrogenase | 5 |
|  |  | PK, pyk; pyruvate kinase | 5 |
|  |  | ACSS; acetyl-CoA synthetase | 5 |
|  |  | glucose-6-phosphate 1-epimerase | 5 |
|  | Citrate cycle  (TCA cycle) | ACLY; ATP citrate (pro-S)-lyase | 6 |
|  |  | LSC1; succinyl-CoA synthetase | 5 |
|  |  | MDH1; malate dehydrogenase | 8 |
|  |  | PDHA, pdhA; pyruvate dehydrogenase | 8 |
|  | Starch and sucrose metabolism | beta-fructofuranosidase | 9 |
|  |  | sucrose-phosphate synthase | 2 |
|  |  | alpha-glucosidase | 6 |
|  |  | beta-glucosidase | 11 |
|  |  | trehalose 6-phosphate synthase | 2 |
|  |  | beta-D-xylosidase 4 | 3 |
|  |  | Pectinesterase | 4 |
|  |  | Polygalacturonase | 6 |
|  |  | glucose-1-phosphate adenylyltransferase | 6 |
|  |  | alpha-amylase | 4 |
|  |  | beta-amylase | 2 |
|  |  | Endoglucanase | 9 |
| Xenobiotics  Biodegradation  and Metabolism | Chloroalkane and chloroalkene degradation | S-(hydroxymethyl)glutathione dehydrogenase / alcohol dehydrogenase | 6 |
|  |  | aldehyde dehydrogenase (NAD+) | 5 |
|  |  | 2-haloacid dehalogenase | 3 |
|  | Toluene and chlorobenzene degradation | catA; catechol 1,2-dioxygenase | 2 |
|  |  | Carboxymethylenebutenolidase | 4 |
|  | Styrene degradation | PHACA; phenylacetate 2-hydroxylase | 1 |
|  |  | FAH; fumarylacetoacetase | 3 |
|  |  | Amidase | 2 |
|  |  | Nitrilase | 1 |
|  |  | phacB; 3-hydroxyphenylacetate 6-hydroxylase | 1 |
|  | Dioxin degradation | salicylate hydroxylase | 6 |
|  | Naphthalene degradation | salicylate hydroxylase | 6 |
|  |  | S-(hydroxymethyl)glutathione dehydrogenase / alcohol dehydrogenase | 6 |
|  | Polycyclic aromatic hydrocarbon degradation | salicylate hydroxylase | 6 |
|  |  | unclassified | 8 |
|  | Metabolism of xenobiotics by cytochrome P450 | GST; glutathione S-transferase | 12 |
|  |  | S-(hydroxymethyl)glutathione dehydrogenase / alcohol dehydrogenase | 6 |
|  |  | AKR7; aflatoxin B1 aldehyde reductase | 1 |
